# Abundance and Survival of Pacific Humpback Whales in a Proposed Critical Habitat Area

**DOI:** 10.1371/journal.pone.0075228

**Published:** 2013-09-11

**Authors:** Erin Ashe, Janie Wray, Christopher R. Picard, Rob Williams

**Affiliations:** 1 Sea Mammal Research Unit, University of St. Andrews, St. Andrews, Fife, Scotland, United Kingdom; 2 Oceans Initiative, Pearse Island, BC Canada; 3 North Coast Cetacean Society, Hartley Bay, BC Canada; 4 Gitga'at Lands and Marine Resources Department, Hartley Bay, BC, Canada; McGill University, Canada

## Abstract

Humpback whales (*Megaptera novaeangliae*) were hunted commercially in Canada's Pacific region until 1966. Depleted to an estimated 1,400 individuals throughout the North Pacific, humpback whales are listed as Threatened under Canada's Species at Risk Act (SARA) and Endangered under the US Endangered Species Act. We conducted an 8-year photo-identification study to monitor humpback whale usage of a coastal fjord system in British Columbia (BC), Canada that was recently proposed as candidate critical habitat for the species under SARA. This participatory research program built collaborations among First Nations, environmental non-governmental organizations and academics. The study site, including the territorial waters of Gitga'at First Nation, is an important summertime feeding destination for migratory humpback whales, but is small relative to the population's range. We estimated abundance and survivorship using mark-recapture methods using photographs of naturally marked individuals. Abundance of humpback whales in the region was large, relative to the site's size, and generally increased throughout the study period. The resulting estimate of adult survivorship (0.979, 95% CI: 0.914, 0.995) is at the high end of previously reported estimates. A high rate of resights provides new evidence for inter-annual site fidelity to these local waters. Habitat characteristics of our study area are considered ecologically significant and unique, and this should be considered as regulatory agencies consider proposals for high-volume crude oil and liquefied natural gas tanker traffic through the area. Monitoring population recovery of a highly mobile, migratory species is daunting for low-cost, community-led science. Focusing on a small, important subset of the animals' range can make this challenge more tractable. Given low statistical power and high variability, our community is considering simpler ecological indicators of population health, such as the number of individuals harmed or killed each year by human activities, including ship strikes and entanglement in fishing gear.

## Introduction

Humpback whales (*Megaptera novaeangliae*) were hunted in Canada's Pacific Region until 1966 [1], [2]. Commercial whaling brought the population of humpback whales in the entire North Pacific from something like 15,000 whales down to 1,400 whales, although there is great uncertainty associated with estimates of abundance at both the population's peak pre-exploitation and its most depleted size [3], [4]. The Committee on the Status of Endangered Wildlife in Canada (COSEWIC) proposed that the population be listed as Threatened, based on low observed densities of humpback whales in British Columbia (BC), as well as vulnerability to human impacts resulting from the whales' strong site fidelity and their propensity to be struck by ships or entangled in fishing gear [5]. Humpback whales were listed as Endangered under the US Endangered Species Act and Threatened under Canada's Species at Risk Act (‘SARA’) [2]. Recent work on the species has revealed strong signs of recovery and the species is now thought to number 21,808 (CV = 0.04) animals in the North Pacific as a whole [4]. This number is thought to exceed some estimates of pre-exploitation abundance, leading to the question of whether humpback whales in Canada's Pacific region are still recovering or completely recovered [6]. COSEWIC has recommended that the population be downlisted to “Special Concern”, and the regulatory agency (Fisheries and Oceans Canada, “DFO”) currently seeks feedback on this proposed downlisting.

The aim of the 2003 SARA listing was to prevent humpback whales from becoming extirpated from Canadian Pacific waters by managing human activities in a way to allow for the whales' recovery. This overarching objective, namely preventing extirpation, is achieved by incorporating the “best available science” into recovery strategy and action plans, for which Fisheries and Oceans Canada (DFO) is the lead agency. Specifically, these plans prohibit human activities that threaten listed species or their critical habitat, and promote stewardship of critical habitat. The draft Recovery Strategy for humpback whales in British Columbia (BC) notes the whales' vulnerability to ship strike, oil spills, entanglement in fishing gear and sensitivity to underwater noise, and calls for studies to assess population health and threats to recovery throughout their range [2]. Appropriate conservation status assessment and recovery planning hinge on good information about population structure, abundance and trends [7], but also on information on individual health and fitness, such as reproductive output and average probability of adult survival (i.e., ‘survivorship’) [8]. Humpback whales in BC appear to consist of two management units, one off the north coast and another off southwestern Vancouver Island [9]. The species is known to show strong site fidelity to local feeding grounds, and this has been documented in BC [9]. The whales that feed in BC spend their winters in a number of mating and calving grounds, including Hawaii, Mexico and Japan [2]. Whales that were seen on BC's north coast (including northern Vancouver Island) were far more likely to be resighted in Hawaii than Mexico or Japan [2], [9], and Canada is currently evaluating whether to treat whales from these two regions as separate stocks for the purposes of conservation and management. Survivorship estimates have been generated for the pooled set of humpback whales that ever pass through Canada's Pacific waters, [2], [9], but if the whales that use north coast waters warrant designation as a separate management unit, demographic data are not available for that unit alone. Survivorship is a useful parameter to measure in order to identify whether a population is threatened by human activities, and an important metric to monitor through time to evaluate whether mitigation and management actions are achieving the desired effect.

The emphasis in Canadian policy on the “best available science” creates an opportunity for the wider research community (e.g., non-governmental organizations (NGOs), First Nations, academia and independent scientists) beyond governmental regulatory agencies to advance our knowledge on imperilled species and participate in the process of endangered species recovery. This approach has been referred to generically as “participatory research” [10], which informs Canadian decision-making along a spectrum ranging from using traditional ecological knowledge as one of many forms of information to guide environmental assessments to formal co-management of natural resources. A major problem with participatory research, though, is the potential for scale mismatch [11]. Endangered species listing and habitat protection decisions are typically made at the national, regional or international scale, whereas funding for the non-governmental sector to engage in field research is usually only at the local scale. Community-university-NGO partnerships play an important role in filling in data gaps in this region [e.g., [12], [13]]. Monitoring population recovery of a highly mobile, migratory species can be difficult for researchers conducting low-cost, community-led science. Focusing on a small, important subset of the animals' range can make this challenge more tractable by bringing the scale of the ecological research to a local one.

As part of its Pacific humpback whale recovery strategy, DFO has proposed four areas as candidate critical habitat [2], [14]. One criterion for designating critical habitats within northern BC coast feeding grounds is that inlets are used for specialized “bubble-net” feeding behaviour [14]. Mainland inlets have been somewhat under-represented in habitat studies to date [15]. We conducted a photo-identification study in north coast, mainland inlets using two independent research platforms.

Humpback whales may be facing increasing threats in at least one of their proposed critical habitats in BC. Numerous port facility expansions and new terminal proposals, including numerous crude oil and liquefied natural gas (LNG) export proposals, could substantially increase deep-sea shipping traffic through BC's north and central coast waters. Such developments could exacerbate oil spill, acoustic disturbance, and ship strike risks to humpbacks. In particular, the Gil Island proposed critical habitat area [2], [14], where our work was conducted, spatially corresponds with all shipping routes leading to Kitimat, BC port facilities that are currently being considered by regulatory agencies for high-volume crude oil and LNG tanker traffic and other increased shipping activities.

Our study was designed to respond to the vision for humpback whale stewardship articulated by North Coast Cetacean Society and the Gitga'at First Nation. The main scientific objective of our study was to estimate abundance of humpback whales using this study area relative to other important habitats for humpback whales in the northeast Pacific. We aim to provide estimates of abundance and survivorship of humpback whales to guide effective management actions, if needed, to mitigate threats to humpbacks that use the area.

## Methods

### Study Area

#### Photo-identification

**Figure 1:**
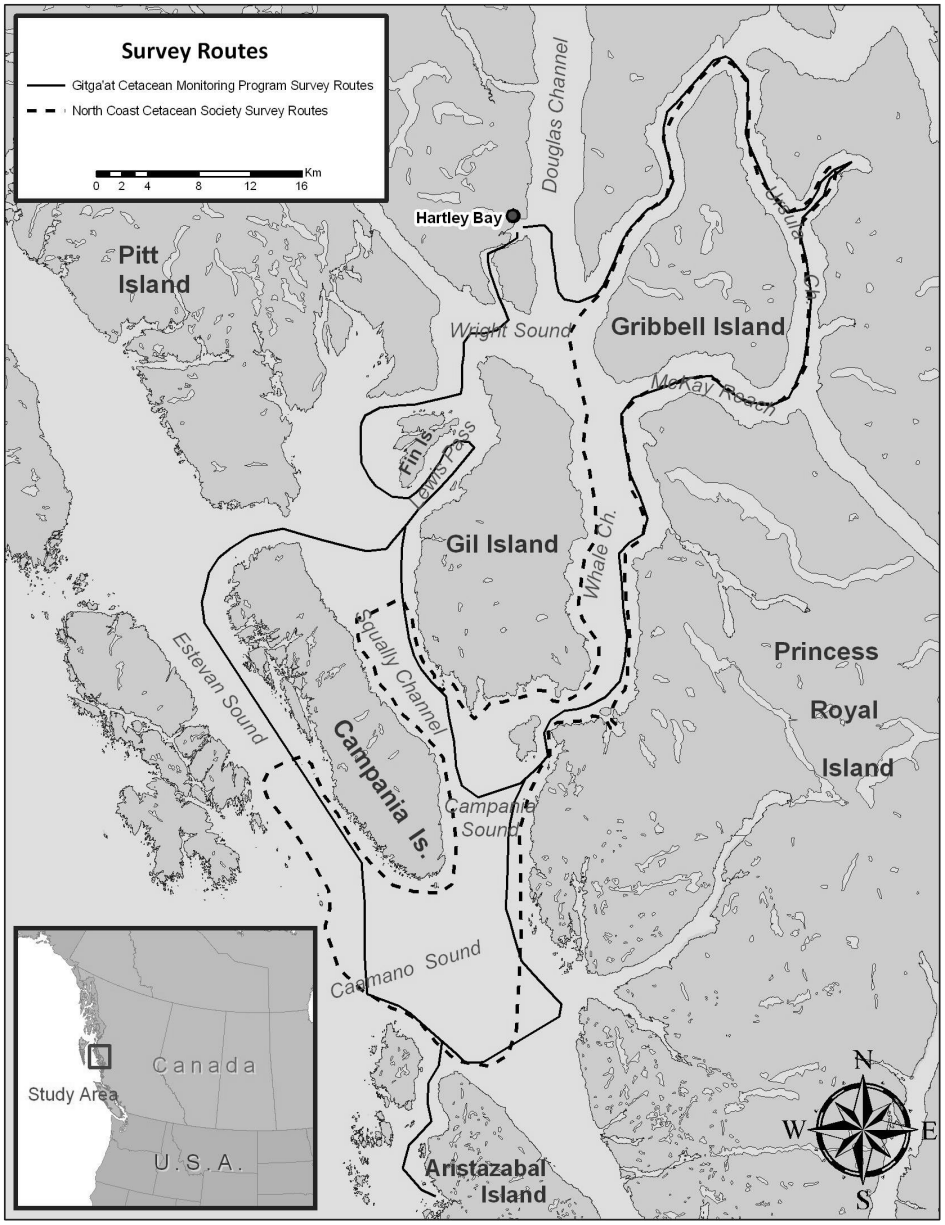
Map of study area and a typical survey route followed by Gitga'at (solid line) and Cetacealab (dashed line). The outer route (solid line, westernmost boundary) shows the route followed when weather conditions allowed observers to search for whales in exposed waters, while the inner line shows the route that would be explored when weather conditions were limiting.

Data were collected under photo-ID license MML 2006-12/SARA-39(A) issued by Fisheries and Oceans Canada. Permits are not required for data collection through hydrophone monitoring. Vessel-based photo-identification surveys were conducted independently off the central coast of BC by two research groups: North Coast Cetacean Society (referred to subsequently as “Cetacealab”); and the Gitga'at Lands and Marine Resources Department (referred to subsequently as “Gitga'at”). Surveys were conducted as weather permitted throughout the year from April to November (with occasional trips in February, March and December), from 2004 to 2011. Typical survey routes for the two groups are shown in Figure 1. All photographs were combined into a single dataset for generating encounter histories (below).

The overarching objective of our field efforts was to collect as many high-quality photographs of individually recognizable humpback whales as possible within the study area (referred to subsequently as “Gil Island waters”) from Estevan Sound in the west to Ursula Channel in the east (Figure 1). One 27′ and one 18′ boat were used to conduct the surveys. A total of 374 photo-identification surveys conducted over 47 months resulted in a catalogue of 177 high-quality, unique identifications of individual humpback whales. In addition, observers were also cued to humpback sightings from three other sources: (1) an informal sightings network including local fishermen and tourism operators who reported humpback and killer whale sightings over VHF radio; (2) an array of hydrophones monitored for vocalizing humpback whales; and (3) visual monitoring from the land-based Cetacealab facility on the south end of Gil Island. When humpback whales were detected, all individuals were counted, location and behaviour noted, and photographs of the underside of their tail flukes were collected, following well-established protocols established for this species (e.g., [6]). All photographs were taken with a standard SLR camera with a telephoto lens.

#### Grading photographs and identifying individuals from natural markings

At the end of each survey, all digital photographs were copied onto a computer. The single best photograph taken of each individual whale on that day was selected, and used to match and identify the humpback whale against a photographic catalogue maintained by our colleagues with the Cetacean Research Program of Fisheries and Oceans Canada (DFO). If an individual whale was not found in the DFO catalogue, it was given a temporary identifier until a BC identifier was assigned by our colleagues at DFO.

Each photograph was graded for photographic quality and distinctiveness of the animal (Table S1), because heterogeneity is introduced by retaining poor-quality photographs, especially of distinctive animals [16]. Incorrect identification can lead to either false positives (which causes negative bias in the abundance estimate) or false negatives (which leads to positive bias in the abundance estimates) [17], [18]. Therefore, all photographs were assigned a score for photographic quality by one of us (JW), following the protocols developed for the SPLASH project [4], [6]. Only photographs of quality 1–3 were used in the analyses (see Table S1 for definitions of quality scores).

### Estimating survival, abundance and temporary emigration

Encounter histories were generated for all uniquely identifiable individuals from good quality photographs. These encounter histories were used to estimate adult (i.e., non-calf) survival and abundance through capture-recapture analysis. Calves were omitted from the analysis.

### Abundance

The 8 years of photo-ID data were used to estimate abundance [19], [20]. Encounter histories were used to estimate the number of individual dolphins, 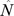 , for pairs of years using the two-sample Chapman modification to the Peterson estimator for small sample size (Equation 1) [19], [20]. 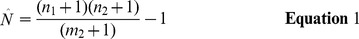 where


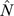
 = abundance estimate; estimate of population size.


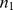
 = the number of individuals detected during the first sampling occasion.


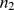
 = the number of individuals detected during the second sampling occasion.


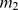
 = the number of individuals re-sighted. That is, the number of marked animals captured during the second sampling occasion that were also captured during the first sampling occasion.

The assumptions of the Chapman estimator are that all animals have an equal probability of being captured, that the individual marks do not change between years, that the marks are correctly identified and recorded, and that the population is closed to births and deaths between years. Chapman estimates were calculated for 7 pairs of years (2004–2005, 2005–2006, 2006–2007, 2007–2008, 2008–2009, 2009–2010, and 2010–2011).

### Survival

Photo-identification data were compiled into an encounter history matrix in which each individual is represented with a row, and columns denote sampling occasion. Captures were represented with a 1 and non-captures with a 0 for each sampling occasion.

Apparent survival rate estimates (Φ) and capture rates (*p*) for well-marked, adult (or at least non-calf) humpback whales were calculated using Cormack-Jolly-Seber (CJS) [21], [22], [23] open population models [24] within Program MARK version 7.1 (http://www.cnr.colostate.edu/~gwhite/mark/). Each of the models, developed independently, employed a time-dependent approach to estimating survival (Φ) and probabilities of recapture (*p*) using capture-recapture data from a particular population or group of animals [8]. CJS is the most general form of survival estimation and provides estimates of Φ and *p*. Survival estimates are not considered estimates of true survival, but rather an estimate of apparent survival as rates of emigration and immigration are not taken into account. The general CJS model can be modified and re-parameterised to include models that estimate constant survival, Φ(.), time varying survival, Φ(t), constant capture probability *p*(.), time varying capture probability *p*(t) and several additional iterations with covariates such as effort and environmental conditions [8].

*CJS model assumptions*:

1. Every marked individual dolphin present in the population at the initial sampling occasion (time *i*) has an equal recapture probability (*p*),
2. Every marked individual dolphin immediately following time (*i*) has an equal survival probability to time (*i*+1),
3. Individual, natural marks are not lost,
4. The duration of a sampling occasion is negligible with respect to the time between sampling occasions, that is, between occasion (*i*) and (*i*+1).

Survival estimates were calculated using year as the sampling unit. In total, 8 years were included. No attempt was made to partition the data by presumed sex. In our case, we restricted our analyses to data collected during sightings surveys from July to September (2004-2011), when most of the animals were expected to be on their summertime feeding ground destination (i.e., rather than migrating through the study area) [4].

A general model was fitted to the data and goodness of fit testing was carried out to assess model fit. The GOF procedure gives an estimate of overdispersion (c-hat) [25] as well as test statistics [8]. There are 4 tests that generate GOF test statistics. TEST3 tests for differences in survival among individuals, and TEST2 tests for heterogeneity among individuals. See [26] for a detailed treatment of the TEST2 and TEST3 procedure. Once a sufficient general model fit was assessed and found to be sufficient, we proceeded to fit the rest of the candidate model set to the data.

Akaike's Information Criteria adjusted for small sample size (AICc) [27] was used to choose the best model among the constructed candidate model set. AIC is an information criterion model selection tool that optimises the balance between model selection and parameter estimation. AIC achieves a compromise between model fit and precision by adding a penalty for each parameter used in the model [27].

### Potential Biological Removal (PBR) Level

We used the Potential Biological Removal (PBR) equation [28] under the US Marine Mammal Protection Act (MMPA) to offer scientific advice to the Gitga'at about the level of harmful human activities that could be sustained by the number of humpback whales that use the study area on average. The PBR equation offers a simple way to estimate the maximum number of animals that may be removed from or seriously harmed in a marine mammal population through human activities, while still allowing that population to reach or maintain its so-called optimum sustainable population. The whales using the study area do not comprise a biologically discrete population, so this calculation is meant only to provide rough, rule-of-thumb guidelines. The data demands of PBR are modest [Equation 1], and require information only on: minimum population size (*N_min_*); one-half the maximum theoretical growth rate of the population at small population size (*R_max_*); and a recovery factor (*F*, ranging from 0.1 to 1.0) that is set to be more precautionary for endangered populations than healthy ones. We used default values of *F* for threatened (*F* = 0.5) stocks [28], because the population is listed under Canada's Species at Risk Act.


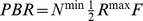
_ [Equation 1]_

## Results

Photo-identification effort was conducted year-round between 2004 and 2011 by two research groups. The distribution of the search effort was constrained to the study area shown in Figure 1. Preliminary analyses revealed that most animals were seen during July, August and September, although several whales were seen in the remaining 9 months of the year.

### Abundance

Abundance estimates for each pair of years are given in Table 1. Our most current (2011) estimate of abundance of humpback whales that use the study area in summer months is 137 (95% CI = 120, 153). Given uncertainty in the abundance estimate, this corresponds to a potential biological removal of 1.29 [Equation 1]. Abundance in the study area has increased each year of the study (2004–2011) (Table 1). Had we used sightings from all months of the year, abundance estimate would have been approximately 20% higher (n1 = 88, n2 = 102, m12 = 54, N = 166).

**Table 1:** Abundance estimates of whales individually identified in each year.

Years	n1	n2	m2	N	Lower 95% CI	Upper 95% CI
2004–2005	24	37	13	68	49	85
2005–2006	37	55	26	79	67	89
2006–2007	55	43	28	85	72	96
2007–2008	43	67	26	111	90	130
2008–2009	67	66	34	130	109	150
2009–2010	66	76	40	126	109	141
2010–2011	76	81	45	137	120	153

### Survival

CJS open recapture models were used to estimate survivorship of adult humpback whales using encounter histories for 177 unique individuals during summer months (July-September) from 2004–2011. Four candidate models of survivorship and capture probability were compared.

Although 3 individuals were seen in each year of the study, 68 individuals were only seen once. The GOF test rejected the CJS model fitted to these data. Since transience may have introduced heterogeneity to the data, the first encounter of every individual was removed from the encounter history before proceeding with model fitting. The CJS model was fit to the reduced data and the GOF test failed to reject the model. The general model, after removing transients, had a median c-hat of 1.35.

Setting both apparent survivorship and capture probability constant provided the best fit to the data (Table 2) and resulted in an estimate of survival of 0.979 (SE = 0.015, CI = 0.914, 0.995).

**Table 2:** Survival models fitted for *Megaptera novaeangliae* 2004–2011.

Model	AICc	ΔAICc	AICc Weights	Model Likelihood	No. Par	Deviance
Ø(.) p(.)	373.7112	0	0.69503	1	2	117.3719
Ø (.) p(t)	375.6449	1.9337	0.2643	0.3803	7	108.8258
Ø (t) p(.)	380.5981	6.8869	0.02221	0.032	7	113.779
Ø (t) p(t)	380.9681	7.2569	0.01846	0.0266	11	105.3971

The model for which survivorship is assumed constant and capture probability is assumed time varying (phi(.)p(t)), also provided reasonably good fit to the data and had only a 1.94 point higher AICc than the best fitting model (Table 2). The point-estimate of adult survival was estimated as 0.975 (SE = 0.0167, 95% CI = 0.910, 0.994) for this model.

## Discussion

The inland waters off the central coast of British Columbia provide important summer feeding habitats and a migratory destination for large numbers of *Threatened* humpback whales. By 2011, 137 (95% CI: 120, 153) humpbacks were estimated to be using our study area. While abundance of humpback whales coastwide in 2009 is unknown, this would represent 8% of a line transect survey-based estimate of 1,310 individuals using north coast waters in 2004-05 [12]. More appropriately, our 2005 abundance estimate represents 6% of the 2005 province-wide estimate. These proportions (6–8%) suggests that a relatively large fraction of BC's humpback whales rely on the waters around Gil Island, given the small size of the study area (6% of abundance found in a study area corresponding to ∼1.5% of the inshore coastal water study area of [12]). This high reliance on relatively small fractions of available habitat has important implications for conservation and management. It lends support to the proposal to designate the current study area as part of the population's critical habitat. In terms of future research, the ability to access and study substantial numbers of BC's humpbacks in one small study area suggests that community-led research may be more tractable for highly mobile and migratory marine species than one might initially think. This also suggests that area-based management for cetaceans can effectively target small areas if these areas are chosen carefully [29]. The corollary to this, though, is that a tendency for animals to be concentrated or aggregated in small areas lends them vulnerable to catastrophic events like oil spills and ship strikes. Critical habitats like the Gil Island waters are therefore a mixed blessing [30] when high densities of whales are found in geographic bottlenecks that also funnel and concentrate shipping traffic. Anthropogenic threats to this must be evaluated not only in terms of the proportion of available habitat that this area represents, but also in terms of its critical importance to large numbers of whales for critical life-history processes. The risk and ecological consequences of an oil spill in this region would increase substantially if proposals were approved to ship large volumes of oil and LNG traffic through the Gil Island waters. Studies in Pacific waters similar to our study area suggest that oil spills can have severe and chronic impacts to cetacean populations and it is uncertain whether affected populations can recover from such perturbations [31]. Our study area has also been identified as candidate critical habitat for northern resident killer whales pending further study [32], and has begun to be recolonized by fin whales in recent years (Cetacealab and Gitga'at, unpublished data). Threats to this habitat therefore have the chance of impacting important habitats for many cetacean species simultaneously.

Our best estimate of apparent survival, which is confounded with permanent emigration, was 0.979 (95% CI: 0.914, 0.995). This point estimate is on the high end of the range of point estimates reported for the species as a whole (ranging from 0.925 to 0.984) [33]. Commercial whaling activities stopped in BC in 1967, and the last humpback whale was killed by BC whalers in 1965 [34]. It is therefore good news that the segment of the population using our study area is growing and adult survival is near the limit that one would expect for this species. That said, although the population is recovering, there is no evidence that it has yet fully recovered to pre-exploitation levels in BC [2], and we do not wish to become complacent. Our future work will continue to monitor whether human-caused mortality is exceeding limits that the population can withstand. The imprecision of abundance and survivorship estimates can make it difficult to evaluate when population declines are occurring [35], and in our case, we also need to be continually aware that we are monitoring only a fraction of the true biological population. If the population shifts distribution in response to shifts in distribution of prey, for example, our surveys alone will not be able to discriminate between distribution shifts and true population declines. We have responded to this in two ways. First, we share identification photos with our colleagues at DFO, who are responsible for monitoring humpback whale populations throughout Canada's Pacific waters, and independent researchers who hold local photo-ID catalogues in other parts of BC. Secondly, our community has adopted a precautionary approach to local resource management that considers how many animals may be killed or harmed each year through human activities. If the humpbacks of the waters around Gil Island formed a biologically discrete population (N = 137, N_min_ = 129, PBR = 1.29), they could withstand the human-caused mortality of approximately one individual each year. In our future work, we aim to assess whether mortality from vessel strikes and entanglement in fishing gear could be causing the death of one humpback whale each year, recognizing that most whale carcasses (whether from natural or anthropogenic causes) go unrecovered [36]. Our community-NGO-First Nations partnership includes a substantial of ocean users in this small, coastal community, so we believe that most fisheries interactions would be reported if detected, whereas vessel strikes from ships transiting the area in rough seas or at night may easily result in a whale death that goes unnoticed, let alone reported. In BC, there is little information on total human-caused mortality in humpback whales. An average of 2.6 humpbacks are reported to be involved in vessel collisions each year, and 1.8 whales per year are involved in fishing gear entanglement, but only a fraction of these interactions are thought to result in mortality or serious injury [2]. Of course, not all incidents are reported. Humpback whales were the most commonly reported cetacean involved in vessel strikes in BC [2]. As a minimum start, we intend to continue our photo-ID work to examine individuals for scars that indicate entanglement or propeller wounds [37], [38]. Next, a quantitative risk assessment is needed to evaluate whether increasing shipping developments, such as proposed oil and LNG tanker traffic, in the Gil Island waters would exacerbate any effects of human activities on humpback whale survival. Given the recognized importance of this habitat to Canada's Pacific population of humpback whales, it is important to continue to monitor survivorship of humpbacks in the region over time, rather than to assume that abundant populations are healthy populations. Our study area was identified in previous analyses as an area of elevated risk of ship strike [39], but that analysis was based on whale abundance in 2004–2006, and our results show that the local population has roughly doubled since 2004 and industrial developments are dramatically changing shipping patterns in the study area.

A future direction of our research is to begin to quantify the sublethal effects of human activities on humpback whales in our study area. The waters around Gil Island are thought to be among the quietest in Canada's Pacific region [40]. Our study area supports a large and growing tourism industry, and repeated disturbance can affect behaviour and activities of humpback whales [41]. An increase in the cumulative impact of stressors that humpback whales experience on feeding grounds could carry costs to substantial fractions of the population. Moreover, habitat loss in BC would impact humpback whales at a particularly vulnerable life-history phase. Humpback whales undergo one of the longest migrations of any mammal [42], therefore anthropogenic activities affecting humpback whales on BC's feeding grounds would impact individuals at a point when they have gone several months without feeding, and may lack resilience to cope with additional human-caused stressors. It is hoped that our information on abundance and survivorship can form a baseline against which future trends can be measured.

## Supporting Information

Table S1 Photographic quality grading description (after Calambokidis et al. 2008).
(DOCX)

## References

[1] Clapham PJ, Leatherwood S, Szzepaniak I, Brownall RLJ (1997) Catches of humpback and other whales from shore stations at Moss Landing and trinidad, California, 1919–1926. Marine Mammal Science 13: 368–394.

[2] Fisheries and Oceans Canada (2010) Recovery Strategy for the North Pacific Humpback Whale (Megaptera novaeangliae) in Canada [DRAFT]. In: Canada FaO, Ottawa: Government of Canada. x+51.

[3] Calambokidis J, Barlow J (2004) ABUNDANCE OF BLUE AND HUMPBACK WHALES IN THE EASTERN NORTH PACIFIC ESTIMATED BY CAPTURE-RECAPTURE AND LINE-TRANSECT METHODS. Marine Mammal Science 20: 63–85.

[4] Barlow J, Calambokidis J, Falcone EA, Baker CS, Burdin AM, et al (2011) Humpback whale abundance in the North Pacific estimated by photographic capture-recapture with bias correction from simulation studies. Marine Mammal Science 27: 793–818.

[5] Baird RW (2003) Update COSEWIC status report on the humpback whale Megaptera novaeangliae in Canada. Ottawa. 1–25.

[6] Calambokidis J, Falcone EA, Quinn TJ, Burdin AM, Clapham PJ, et al. (2008) SPLASH: Structure of Populations, Levels of Abundance and Status of Humpback Whales in the North Pacific. Olympia, WA: Cascadia Research. 1–57.

[7] Gerber LR, DeMaster DP (1999) A quantitative approach to Endangered Species Act classification of long-lived vertebrates: application to the North Pacific Humpback whale. Conservation Biology 13: 1203–1214.

[8] Lebreton J-D, Burnham KP, Clobert J, Anderson DR (1992) Modeling Survival and Testing Biological Hypotheses Using Marked Animals: A Unified Approach with Case Studies. Ecological Monographs 62: 67–118.

[9] Rambeau AL (2008) Determining abundance and stock structure for a widespread migratory animal: the case of humpback whales (Megaptera novaeangliae) in British Columbia, Canada. Vancouver: University of British Columbia. 70.

[10] Berkes F, Mathias J, Kislalioglu M, Fast H (2001) The Canadian Arctic and the Oceans Act: the development of participatory environmental research and management. Ocean & Coastal Management 44: 451–469.

[11] Stevens C, Fraser I, Mitchley J, Thomas M (2007) Making ecological science policy-relevant: issues of scale and disciplinary integration. Landscape Ecology 22: 799–809.

[12] Williams R, Thomas L (2007) Distribution and abundance of marine mammals in the coastal waters of British Columbia, Canada. JOURNAL OF CETACEAN RESEARCH AND MANAGEMENT 9: 14.

[13] Williams R, Thomas L (2009) Cost-effective abundance estimation of rare animals: Testing performance of small-boat surveys for killer whales in British Columbia. Biological Conservation 142: 1542–1547.

[14] Nichol LM, Abernethy R, Flostrand L, Lee TS, Ford JKB (2010) Information relevant for the identification of Critical Habitats of North Pacific Humpback Whales (Megaptera novaeangliae) in British Columbia. Ottawa. iv+40 .

[15] Dalla Rosa L (2010) Modeling the foraging habitat of humpback whales. Vancouver: University of British Columbia. 185 .

[16] Wilson B, Hammond PS, Thompson PM (1999) Estimating size and assessing trends of a coastal bottlenose dolphin population. Ecological Applications 9: 288–300.

[17] Friday N, Smith TD, Stevick PT, Allen J (2000) Measurement of photographic quality and individual distinctiveness for the photographic identification of humpback whales, {IMegaptera novaeangliae}. Marine Mammal Science 16: 355–374.

[18] Stevick P, Palsboll PJ, Smith TD, Bravington MV, Hammond PS (2001) Errors in identification using natural markings: rates, sources, and effects on capture-recapture estimates of abundance. Can J Fish Aquat Sci 58: 1861–1870.

[19] Seber GAF (1982) The Estimation of Animal Abundance. New York: Hafner Publ. Co., Inc. 654 .

[20] Hammond PS (1986) Estimating the size of naturally marked whale populations using capture-recapture techniques. Reports of the International Whaling Commission Special 8: 253–282.

[21] Cormack RM (1964) Estimates of Survival from the Sighting of Marked Animals. Biometrika 51: 429–438.

[22] Jolly GM (1965) Explicit estimates from capture-recapture data with both death and immigration - a stochastic model. Biometrika 52: 225–247. 14341276

[23] Seber GAF (1965) A note on multiple recapture census. Biometrika 52: 249–259. 14341277

[24] White GC, Burnham KP (1999) Program MARK: survival estimation from populations of marked animals. Bird Study 46: 120–139.

[25] Burnham KP, Anderson DR (2002) Model selection and multi-model inference: a practical information-theoretic approach: Springer Verlag.

[26] Burnham KP, Anderson DR, White GC, Brownie C, Pollock KH (1987) Design and Analysis Methods for Fish Survival Experiments Based on Release-Recapture. Bethesda, Maryland: American Fisheries Society. x+437 .

[27] Burnham KP, Anderson DR (2003) Model selection and multimodel inference: A practical information-theoretic approach. New York: Springer-Verlag. 488 .

[28] Wade PR (1998) Calculating limits to the allowable human-caused mortality of cetaceans and pinnipeds. Marine Mammal Science 14: 1–37.

[29] Ashe E, Noren DP, Williams R (2010) Animal behaviour and marine protected areas: incorporating behavioural data into the selection of marine protected areas for an endangered killer whale population. Animal Conservation 13: 196–203.

[30] Williams R, Lusseau D, Hammond PS (2009) The role of social aggregations and protected areas in killer whale conservation: The mixed blessing of critical habitat. Biological Conservation 142: 709–719.

[31] Matkin CO, Saulitis EL, Ellis GM, Olesiuk P, Rice SD (2008) Ongoing population-level impacts on killer whales Orcinus orca following the Exxon Valdez oil spill in Prince William Sound, Alaska. Marine Ecology Progress Series 356: 269–281.

[32] Ford JKB (2006) An Assessment of Critical Habitats of Resident Killer Whales in Waters off the Pacific Coast of Canada. Ottawa: Canadian Science Advisory Secretariat. 39 .

[33] Zerbini A, Clapham P, Wade P (2010) Assessing plausible rates of population growth in humpback whales from life-history data. Marine Biology 157: 1225–1236.

[34] Nichol LM, Gregr EJ, Flinn R, Ford JKB, Gurney R, et al (2002) British Columbia commercial whaling catch data 1908 to 1967: A detailed description of the BC historical whaling database. Canadian Technical Report of Fisheries and Aquatic Sciences 2396: 82.

[35] Taylor BL, Martinez M, Gerrodette T, Barlow J, Hrovat YN (2007) LESSONS FROM MONITORING TRENDS IN ABUNDANCE OF MARINE MAMMALS. Marine Mammal Science 23: 157–175.

[36] Williams R, Gero S, Bejder L, Calambokidis J, Kraus SD, et al (2011) Underestimating the damage: interpreting cetacean carcass recoveries in the context of the Deepwater Horizon/BP incident. Conservation Letters 4: 228–233.

[37] Robbins J, Mattila DK (2001) Monitoring entanglements of humpback whales (*Megaptera novaeangliae*) in the Gulf of Maine on the basis of caudal peduncle scarring. Paper presented to the Scientific Committee of the International Whaling Commission. SC/53/NAH25 SC/53/NAH25.

[38] Moore MJ, Barco S, Costidis A, Gulland F, Jepson P, et al (2013) Criteria and case definitions for serious injury and death of pinnipeds and cetaceans caused by anthropogenic trauma. Diseases of aquatic organisms 103: 229–264. 23574708 10.3354/dao02566

[39] Williams R, O'Hara P (2010) Modelling ship strike risk to fin, humpback and killer whales in British Columbia, Canada. Journal of Cetacean Research and Management 11: 1–8.

[40] Erbe C, MacGillivray A, Williams R (2012) Mapping cumulative noise from shipping to inform marine spatial planning. The Journal of the Acoustical Society of America 132: EL423–EL428. 23145705 10.1121/1.4758779

[41] Scheidat M, Castro C, González J, Williams R (2004) Behavioural responses of humpback whales (*Megaptera novaeangliae*) to whalewatching boats near Isla de la Plata, Machalilla National Park, Ecuador. Journal of Cetacean Research and Management 6: 63–68.

[42] Stevick PT, Neves MC, Johansen F, Engel MH, Allen J, et al (2011) A quarter of a world away: female humpback whale moves 10 000 km between breeding areas. Biology Letters 7: 299–302. 20943678 10.1098/rsbl.2010.0717 PMC3061163

