# Supplementary material for: Abundance and Survival of Pacific Humpback Whales in a Proposed Critical Habitat Area
Source: PLoS One. 2013 Sep 11;8(9):e75228. doi: 10.1371/journal.pone.0075228 (PMC3772752; doi:10.1371/journal.pone.0075228)
Supplement: Table S1 — Photographic quality grading description (after Calambokidis et al. 2008). (DOCX) [file pone.0075228.s001.docx]

Supporting Information

Table S1: Photographic quality grading description (after Calambokidis et al. 2008).

| **Score** | **1** | **2** | **3** | **4** | **5** |
| --- | --- | --- | --- | --- | --- |
|  |  |  |  |  |  |
| **Proportion of fluke visible (%)** | 100 | 75-99 | 50-74  (notch visible) | <50 | Left/right side only |
| **Fluke vertical angle** | Perpendicular | Short of perpendicular but no loss in visibility | Short of perpendicular; some loss in quality, ridging easily visible | Low angle, ridging only partially visible | Low angle, ridging and markings not visible/very distorted |
| **Photographer lateral angle** | Directly behind | Not directly behind but minimal distortion | Angled about 45° to side | Angled >45° but markings still visible | Angle so extreme most markings obscured |
| **Focus/**  **Sharpness** | Excellent focus with clear grain | Good focus and grain with only minimal loss in quality | Acceptable focus and grain; some loss in discerning marks and edges | Fair to poor focus in grain with significant loss in clarity | Soft focus/grainy with extreme loss in detail |
| **Lighting/**  **contrast/**  **exposure** | Excellent lighting and contrast, any marks present would be seen | Good but with some loss in contrast on ventral surface | Fair, some marks might not be seen at all but most would likely be visible | Fair to poor with significant backlighting or exposure problems | Poor (e.g. back lit or gray), likely many marks would not be visible |
| **Whale distinctiveness** | Distinctive and numerous scars and markings | Either a few distinctive marks or numerous not very distinctive marks | Marks present but neither distinctive nor numerous | Few faint and not distinctive scars or markings | No marks visible |
| **Overall Photo Quality** | **Excellent**, ratings of 1 for exposure, focus, lateral angle, vertical angle, and percentage of fluke visible. | **Good**, rating of 2 in 1-2 categories, 1 in all the rest Quality of photo does not compromise the reviewer’s ability to identify whale | **Okay**, 1 rating of 3 or 3 ratings of 2, the rest a rating of 1. Identification definitely possible. | **Poor**, rating of 4 for at least one criterion. Identification possible, but only if distinctive markings or features present. Photo editing may be required. | Identification from this photo is not possible unless whale is well known and/or has extremely distinctive markings. |
